# What if Cannabis has a medical relevance in psychiatric disorders?

**DOI:** 10.1192/j.eurpsy.2022.1859

**Published:** 2022-09-01

**Authors:** B. Abassi, E. Khelifa, K. Nourchene, O. Maatouk, L. Mnif

**Affiliations:** 1 Razi, Skolly, Manouba, Tunisia; 2 Razi hospital, Skolly, Tunis, Tunisia; 3 Razi Hospital, F Adult Psychiatry Department, Manouba, Tunisia

**Keywords:** medicinal cannabis, synthetic cannabinoids, PTSD, psychiatric illness

## Abstract

**Introduction:**

Cannabis was used as a medicinal plant in Asia before the Christian era. Nowadays, after 40years of a “war on drugs” with an illegal status, there is a big interest on the use of cannabis in some medical conditions. With more and more users revealing having used this substance to cope with certain psychiatric manifestations, researchers have tried to explore this substance in the psychiatric field where the actual pharmacological treatments and psychotherapy remain ineffective in some cases.

**Objectives:**

To Explore the medical use of cannabis in psychiatric disorders.

**Methods:**

A literature review was based on the PubMed interface and adapted for 2 databases: Science Direct and Google Scholar over the last 10 years.

**Results:**

Giving the interactions between cannabinoids and specific neurotransmitters, it has been suggested that cannabis may have medical effect on some psychiatric illnesses. In this direction, a significant overlap has been demonstrated between PTSD and cannabis use. CBD a non-psychotomimetic cannabinoid, seemed to show promising results as an enhancer of fear extinction and therapeutic consolidation of emotional memories. Military veterans are increasingly using it for reducing induced nightmares although this residual symptom remains difficult to treat. No benefit for improving depression was proved. One isolated study indicated a potential efficacy for cannabinoid combined with terpene in ADHD.

**Conclusions:**

Studies exploring the possibility of using cannabis in the treatment of psychiatric illnesses are promising but it is premature to recommend this drug for the moment especially since it requires gradual titration, regular assessment and precaution in certain diseases.

**Disclosure:**

No significant relationships.

